# Applications of the ultrasound‐guided nerve block technique for nonanalgesic effects

**DOI:** 10.1002/ibra.12061

**Published:** 2022-08-15

**Authors:** Guang‐Ting Zhang, Feng‐Lin Wang, Ying Ran, De‐Xing Liu

**Affiliations:** ^1^ Department of Anesthesiology Affiliated Hospital of Zunyi Medical University ZunYi Guizhou China

**Keywords:** enhanced recovery after surgery, multi‐system disease, nerve block technique, ultrasound

## Abstract

The nerve block technique guided by ultrasound has been able to accurately block tiny nerves throughout the body in recent years. It has been increasingly used to treat multisystem diseases or analgesia in surgical patients, but the latter accounted for the vast majority of cases. The nonanalgesic effect of nerve blocks is also in wide demand. After searching ultrasound‐guided nerve block works on the PubMed database, we systematically summarized the current clinical application of the nerve block technique and the unique role and related mechanism of nerve block in the prevention and treatment of multi‐system diseases or symptoms, including disorders of the circulatory and respiratory systems, postoperative cognitive dysfunction, immune function, posttraumatic stress disorder, and postoperative digestive system, to put forward the potential prospective application in future and serve as a reference for future research of nerve block therapy in these diseases mentioned.

## Graphical abstract

Diagram of the review. POCD, postoperative cognitive dysfunction; PTSD, posttraumatic stress disorder; ERAS, enhanced recovery after surgery; PAH, pulmonary arterial hypertension.

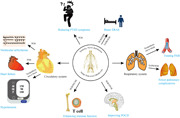

## 1 INTRODUCTION

To achieve the effect of clinical treatment, the nerve block technique where local anesthetics are injected around nerves, such as the nerve trunk, nerve plexus, and sympathetic conducting impulses was performed. At the Eye Conference in Heidelberg, Germany in 1884, Austrian researcher Koller revealed the use of cocaine in ocular surface anesthesia.^1^ Subsequently, Franck found that cocaine could produce “physiological and segmental” anesthetic effects on both sensory and motor fibers, and named the special effect “nerve block” in 1892.^2^ The invention and clinical application of local anesthetics, such as lidocaine, bupivacaine, and ropivacaine have promoted the development of nerve block techniques. Additionally, with the development of ultrasound, the ultrasound‐guided peripheral nerve block was published firstly in 1994, elevating the nerve block technique to a new level of technical application.^3^

With the guidance of ultrasound technology, nerve block techniques, such as splanchnic nerve block (SNB), paravertebral block (PVB), stellate ganglion block (SGB), and even terminal tiny nerve block have been widely performed in recent years. As a result, more and more studies involving nerve blocks have been conducted. In addition to their analgesic effects in patients after surgical procedures, nerve blocks have been reported to have some distinct beneficial roles in preventing and regulating multi‐system disorders or symptoms and enhanced recovery after surgery (ERAS). After searching ultrasound‐guided nerve blocks on the PubMed database and excluding analgesia‐related literature, we found that articles described mainly circulatory, respiratory, postoperative cognitive dysfunction (POCD), immune function, posttraumatic stress disorder (PTSD), and postoperative nausea and vomiting (PONV). So, this study focuses on summarizing the effects of nerve block on treatment and intervention for the aforementioned disorders. We hope this work can inspire doctors and scholars to pay attention to the unique role of nerve block and serve as a reference for future research on the nonanalgesic effect of nerve block technique.

## 2 THE APPLICATION OF THE NERVE BLOCK TECHNIQUE IN THE TREATING CIRCULATORY DISEASES

Circulatory diseases, such as heart failure, malignant arrhythmia, and hypertension are frequently treated primarily with drugs. However, with the rapid development of the nerve block technique, nerve block has gradually been used in the treatment of these diseases in recent years, providing medical staff with another protocol for the treatment of those who have failed in drugs or developed drug resistance.

### 2.1 The therapeutic effect of splanchnic nerve block on heart failure

Common clinical manifestations of heart failure include symptoms, such as fatigue, shortness of breath, and decline in inactivity, as well as signs, such as a decline in cardiac output or cardiac index (CI), and an increment in systemic vascular resistance (SVR). Drugs are conventional therapeutic protocols for heart failure, including angiotensin‐converting enzyme inhibitors, beta antagonists, diuretics, and inotropes. But they have related contraindications and may cause side effects, such as cough, bradycardia, and homeostasis. Therefore, to avoid these side effects of drugs, some experts try to use SNB to treat heart failure.^4^ SNB is defined as the procedure of blocking splanchnic nerve impulse conduction by injecting local anesthetics or nerve‐damaging chemicals into the celiac plexus, which consists of the great splanchnic nerve, the lesser splanchnic nerve, and the smallest splanchnic nerve. In the initial investigation using SNB, Fudim's team used bilateral T11‐T12 injections of 1% lidocaine 15 ml to treat five patients with acute heart failure.^4^ The CI rose 0.62 L/min/m^2^ after 30 min and the SVR dropped 599 dynes s cm^−5^. Following that, they utilized bilateral SNB to treat patients with chronic decompensated heart failure, low mobility and less than 30% of left ventricular ejection fraction (LVEF). Surprisingly, the mean pulmonary capillary wedge pressure (PCWP) decreased by 8 mmHg, the CI increased by 0.62 L/min/m^2^ after 30 min, as well as the 6‐min walking distance increased by 16 m on average after 24 h.^5^ Even though temporary orthostatic hypotension was observed in four individuals, it was cured with mild fluid replacement. On this basis, Fudim conducted a further study, enrolled 19 patients, 15 of whom underwent percutaneous SNB, in which subjects were diagnosed with chronic heart failure with an LVEF of less than 35%, and primary outcomes included mean pulmonary artery pressure (mPAP), PCWP, and exercise capacity. After bilateral SNB with 12 ml ropivacaine of 0.5%, injected into the anterolateral edge of the T12‐L1 vertebral body, mPAP and PCWP decreased by 8.3 mmHg and 9.7 mmHg respectively, and CI increased by 0.4 L/min/m^2^. In addition, 6‐min walking distance, exercise volume, dyspnea, and fatigue all were improved greatly, all of which were statistically significant.^6^

The proposed mechanism of SNB in the treatment of heart failure is that it reduces biventricular preload and afterload, which alleviate heart failure symptoms while increasing cardiac output and activity. Some studies suggested that SNB can improve pulmonary congestion by increasing the storage capacity of the blood vessel bed of visceral volume, which can reduce the visceral sympathetic nerve tension and dilate the internal diameter of blood vessels of sympathetic sensitive viscera (liver, spleen, and intestinal tract, etc.).^7^, ^8^ Briefly, heart failure symptoms, such as weariness and shortness of breath were alleviated, following the volume of abdominal visceral blood growing while the volume of intrathoracic blood dropped. In the effect on redistribute blood volume, a nerve block may be more effective than drugs.

### 2.2 The efficacy of sympathetic nerve block in the treatment of refractory arrhythmias

**Figure 1:**
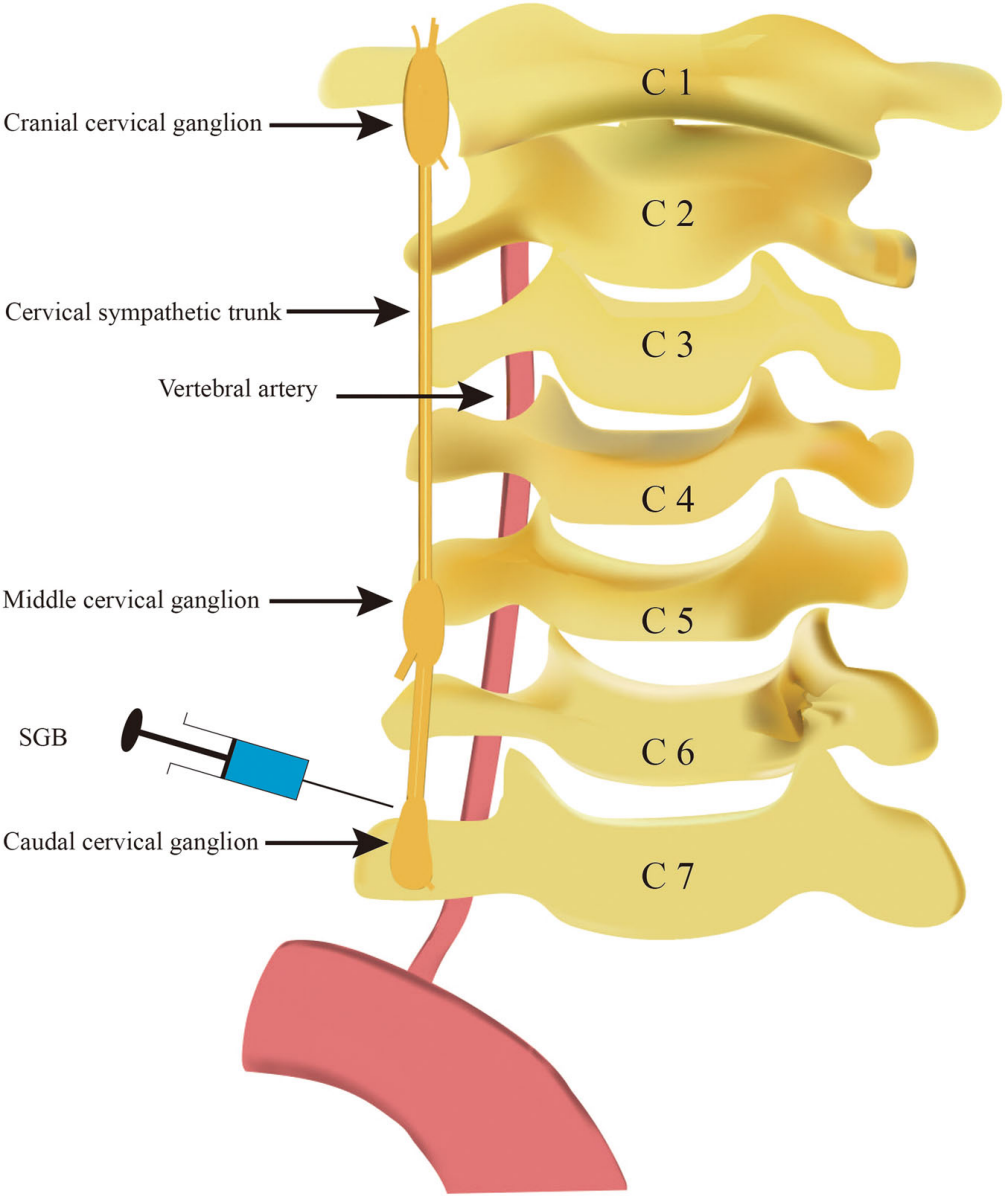
Stellate ganglion block (SGB) flowchart [Color figure can be viewed at wileyonlinelibrary.com]

Severe ventricular arrhythmias mainly have ventricular tachycardia (VT), ventricular flutter, and ventricular fibrillation (VF) with variable clinical manifestations. Patients with disorders may be asymptomatic or have clear palpitations or amaurosis, and even sudden cardiac death. At present, antiarrhythmic drugs and radiofrequency catheter ablation are the main treatment methods. However, some refractory arrhythmias have a low response to pharmacological therapy and reoccur repeatedly after catheter ablation. Sympathetic nerve block has been demonstrated in studies to treat refractory ventricular arrhythmias and reduce the incidence of recurrence.^9^, ^10^ A case showed that bilateral T2 and T3 thoracic sympathetic nerve block could mediate VT after antiarrhythmic medicines had little effect.^11^ In another study, three patients, with VT/VF caused by pacemakers, were given bilateral PVB after amiodarone, propafenone, and metoprolol, even if catheter ablation failed. And participants obtained a significant reduction in the burden of refractory ventricular tachyarrhythmias after bilateral PVB.^12^ SGB, as a sympathetic nerve block (Figure 1), also plays an important role in treating VT and/or VF. In a trial, ultrasound‐guided bilateral SGB can reduce safely and effectively the burden of ventricular arrhythmias, with approximately one in two patients exhibiting complete suppression of VT or VF for 48 h.^13^ Similarly, single left SGB also can alleviate effectively VT/VF and be used as an adjuvant to conventional therapy in individuals with refractory ventricular arrhythmias.^14^ In another study, 30 patients with refractory ventricular arrhythmias were performed SGB after treating by more than two types of antiarrhythmic medicines, with 15 patients receiving left SGB and the other 15 bilateral SGB, which showed that left SGB and bilateral SGB have equal efficacy in attenuating electrical storm.^15^ And they may be used to stabilize ventricular rhythm in individuals who have failed to respond to other therapies.^15^ Furthermore, although local anesthetics maintain a block length of 3–7 h, the inhibitory impact on arrhythmia can extend for several days.^12^, ^16^ As a result, the procedure might prevent successfully patients from being performed frequently nerve block and nerve resection with adverse denervation responses. The effectiveness of SGB can be utilized as an alternative method for treating ventricular arrhythmia in individuals who have failed in other treatments and there is no difference in the effect of left and bilateral SGB on malignant arrhythmias. So, the patients with ventricular arrhythmias will be treated with a sympathetic nerve block, especially SGB.

### 2.3 The impact of sympathetic nerve block on the therapy of hypertension

There are many causes of hypertension, increased sympathetic tone is one of them.^17^ Therefore, reducing sympathetic tone can alleviate the progress of hypertension. However, antihypertensive drugs have a limited impact on lowering the sympathetic tone. Nerve blocks may have better potency in the function. The sympathetic nerve block was reported to cure refractory hypertension in 1947,^18^ which greatly accelerated the progress of hypertension treatment. Furthermore, renal sympathetic nerves are unusually tight in people with renal hypertension, causing the renin‐angiotensin‐aldosterone system to become hyperactive. At this time, a sympathetic nerve block can improve the hypertensive renal sympathetic tone, renal artery stenosis, and renal ischemia in patients with renal hypertension.^19^, ^20^ SGB also has an excellent effect on reducing hypertension during surgery.^21^ However, a coin has two sides, SGB can lead to hypertension and even hypertensive emergencies, there is a description that the local anesthetic penetrates the carotid sheath and blocks vagus nerves, such as the sinus nerve at the carotid sinus or the glossopharyngeal nerve, causing sympathetic nerves to become hyperactive and resulting in hypertensive emergencies.^22^ So, clinicians must learn the anatomy of the stellate ganglion and be proficient in ultrasound technology to decrease the usage of local anesthetics and avoid complications.

## 3 THE APPLICATION OF THE NERVE BLOCK TECHNIQUE IN THE TREATING RESPIRATORY DISEASES

In recent years, the nerve block has been utilized to treat lung diseases, especially pulmonary arterial hypertension (PAH). Nerve blocks also play a role in reducing pulmonary complications after surgery, they can promote the development of ERAS.

### 3.1 The function of cervical sympathetic nerve block in reducing pulmonary arterial hypertension

**Figure 2:**
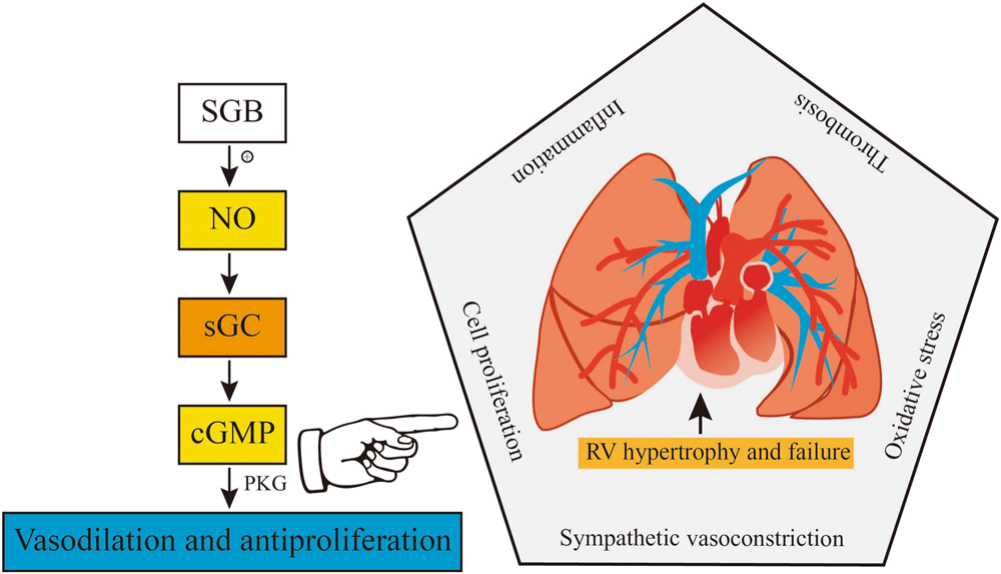
Mechanism of stellate ganglion block (SGB) in the treatment of pulmonary arterial hypertension. cGMP, cyclic guanosine monophosphate; NO, nitric oxide; PKG, cGMP‐dependent protein kinase; RV, right ventricle; sGC, soluble guanylate cyclase. [Color figure can be viewed at wileyonlinelibrary.com]

PAH is characterized by progressive pulmonary vascular remodeling, persistent elevation of mPAP, and pulmonary vascular resistance, resulting in right ventricular hypertrophy and dysfunction, ultimately leading to mortality. There are many factors in the initiation and progression of PAH, including sympathetic anxiety, vasoactive chemical imbalance, cell proliferation, vascular remodeling, inflammation, thrombosis and oxidative stress, and so on.^23^ Atherosclerosis, left heart failure, pulmonary disease or hypoxia, chronic thromboembolic illness, and unknown or multifactorial PAH are the five subtypes of PAH.^24^ Currently, vasodilators such as phosphodiesterase inhibitors and endothelin receptor blockers are used to treat PAH, but their therapeutic benefits are modest. Reed reported that cervical sympathetic nerve block can boost the generation of local nitric oxide (NO),^25^ which has a vasodilator effect. And PAH can lead to increase expression of arginase, inducing decreased production of NO.^26^ Furthermore, the team led by Na found that blocking rat carotid sympathetic ganglia can inhibit arginase activity to increase NO production to reduce PAH for monocrotaline‐induced pulmonary hypertension in rats.^27^ NO can treat PAH through the NO‐sCG‐cGMP pathway.^28^ Therefore, the cervical sympathetic nerve block is a choice for therapy of PAH. The mechanism of the effect of the cervical sympathetic nerve block is shown in detail in Figure 2. Since the pulmonary sympathetic innervation mainly comes from the middle and lower cervical sympathetic chain (C7‐T8) and the sympathetic nerve is dominant in the pulmonary artery innervation.^29^ And the stellate ganglion is formed by the inferior cervical ganglion and the first thoracic ganglion.^30^ So, we speculate that SGB also affects reducing PAH. There is a broad prospect of nerve block in the treatment of PAH, but a large sample, multicenter experiments need to be performed.

### 3.2 The effect of nerve block on improving postoperative pulmonary complications

Many patients after major surgery are at risk for pulmonary complications as a result of prolonged bed rest following surgery, which prolongs postoperative recovery time and increases hospitalization expenditures, which are not conducive to the development of ERAS. However, nerve blocks have been proven in trials to lower the incidence of postoperative pulmonary complications in patients.^31^, ^32^ Intercostal nerve block has been shown in studies to lower the incidence of pulmonary complications such as atelectasis in patients who have undergone upper abdominal incision and thoracotomy surgery.^32^, ^33^ In terms of the effect of nerve block on lung function, Basaran et al. performed subcostal transversus abdominis plane block (TAPB) in patients undergoing laparoscopic cholecystectomy and discovered that the degrees of reduction in forced expiratory volume 1 (FEV1) and forced vital capacity (FVC) were significantly less than in the nonblocking group in the 24 h after surgery, implying that TAPB plays an important role in improving respiratory function in patients after surgery.^34^ Similarly, thoracic paravertebral nerve block (TPVB) can also improve lung function indicators like FEV1, FVC, and forced expiratory flow in individuals who have had thoracoscopic surgery and promote postoperative recovery, as well as shorten the length of hospital stay.^35^

Nerve block also can alleviate acute lung injury in rats or patients. Chen^36^ shared a finding that SGB can reduce acute lung injury in septic rats after making cecal ligation and perforation, and suggested that SGB may suppress the inflammatory response by lowering myeloperoxidase (MPO) activity and NF‐κB signal transduction, thereby curing sepsis Acute lung injury in rats. Another research indicated that SGB can reduce lung inflammation by inhibiting the production of proinflammatory cytokines, such as interleukin‐1 (IL‐1), IL‐6, and tumor necrosis factor‐α.^37^ Furthermore, SGB has no adverse effect on pulmonary resistance, bronchial and pulmonary blood flow, or arterial blood gas levels.^37^ So SGB is thought to be safe for patients with acute lung damage.^37^ The effect of a TPVB on lung injury is also beneficial. According to a randomized controlled trial (RCT) including 120 patients with lung cancer having thoracoscopic surgery, TPVB can lower lung injury scores, apoptosis, and inflammation to prevent lung damage.^38^ Furthermore, TPVB can significantly improve the mobility of patients following thoracoscopic radical resection of lung cancer, which is beneficial to their rapid recovery after surgery.^31^ Additionally, complications, such as bradycardia or hypotension, local anesthetic poisoning, pleural puncture, and pneumothorax from TPVB are very low and worthy of clinical application.^39^, ^40^

## 4 THE APPLICATION OF THE NERVE BLOCK TECHNIQUE IN ALLEVIATING POSTOPERATIVE COGNITIVE DYSFUNCTION

POCD, a central nervous system complication, often occurs in elderly patients several days to several weeks after general anesthesia surgical operation, and it may cause permanent cognitive impairment.^41^ Mild manifestation is cognitive function impairment, and severe cases may show personality changes and a decline in quality of life after surgery, and even a rise in the mortality of elderly patients.^42^ POCD after general anesthesia and major surgery is related to an increase of β‐amyloid (Aβ) protein and tau ratio,^43^ serum S100 protein,^44^ and even changes in the hippocampus ultrastructure.^45^ When central nerve cells are injured, these proteins will increase release.^43^, ^46^ On the other hand, POCD has been linked to surgical stress and anomalies in glutamate neurons in a recent study.^47^ Studies showed that nerve blocks can improve POCD in patients after the surgical procedure.^48^, ^49^

Shi's study on elderly patients undergoing hip replacement suggested that epidural block can reduce plasma Aβ and Tau in patients to reduce the incidence of POCD compared with general anesthesia.^50^ But another study had shown that TPVB has a better effect on POCD than epidural blockade, owing to TPVB can reduce S100 protein,^51^ as well as reduce plasma Aβ and Tau proteins. Furthermore, with TPVB's targeted block on the somatosensory nerves on the surgical side, the pain signals conducted by sympathetic nerves are blocked more thoroughly. As a result, TPVB outperforms epidural block in terms of reducing detrimental stress and inflammation while maintaining the internal environment's stability.^52^

The SGB also has a potential impact on alleviating POCD. In Zhang's research,^53^ elderly patients undergoing coronary artery bypass graft under cardiopulmonary bypass (CPB) who were performed right SGB before anesthesia induction, improving cerebral oxygen metabolism. After right SGB, the right side regional cerebral oxygen saturation (rSO_2_) was significantly higher than the left side, and the left side rSO_2_ of participants after SGB was significantly higher than that of the control group.^53^ Simultaneously, the incidence of POCD in the SGB group was significantly lower than that in the control group within 7 days after surgery.^53^ According to some studies, SGB blocks the sympathetic nerve trunk to relieve intracranial vasospasm, dilate blood vessels, and reduce vascular resistance, thereby improving blood circulation and the balance of oxygen supply and demand in the brain, and ultimately reducing the incidence of POCD in the elderly patients.^54^ To summarize, we believe that SGB improves POCD by enhancing perioperative cerebral oxygen metabolism and alleviating surgical trauma, hence stabilizing cerebral circulation and the balance of cerebral oxygen metabolism, as well as reducing the stress response and nerve cell damage.

However, there are still many gaps in the research on the effect of nerve blocks on POCD in the elderly population. A great number of multi‐center and large‐sample clinical research are bound to be carried out to clarify the mechanism of nerve block improving POCD in elderly patients, reduce the incidence of POCD, and improve the quality of life of elderly patients following surgery.

## 5 THE APPLICATION OF THE NERVE BLOCK TECHNIQUE IN IMPROVING IMMUNE FUNCTION

For the recovery of many diseases, a high level of immunological activity is critical. Tumors, surgery, and anesthesia, among other things, can lower patients' immunity, making it difficult for them to recover. Because of the low degree of immunity following tumor surgery, leftover tumor cells may travel far. To promote the development of ERAS, nerve blocks have been used to manage perioperative immune dysfunction in patients after surgery or with immune diseases.^55^

### 5.1 Effect of nerve block on immune function after cancer surgery

The thoracic nerve block II (PECS II block) was performed based on modifying the thoracic nerve block I by Blanco.^56^ Local anesthetics were injected into the gaps between the pectoralis major and minor, as well as between the pectoralis minor and the serratus anterior, at the third intercostal. It has a good blocking effect on the lateral cutaneous branches of the intercostal nerves from the 2nd to the 6th, the intercostal brachial nerve, the medial and lateral thoracic nerves, and the long thoracic nerve, as well as the dorsal nerves, making it particularly useful for axillary lymph node dissection and wide excision surgeries. Although surgical resection is the conventional treatment option for early breast cancer, it is still possible for cancer cells to recur or spread to distant sites due to immunodeficiency, which is caused by surgical trauma, volatile anesthetics, and high opioid consumption, reducing the number of natural killer (NK) cells and their activity.^57^, ^58^ NK cells, which are a key aspect of innate immunity, can defend the body from tumor cell invasion and spread. Trastuzumab is a routinely used adjuvant therapy for Her2‐positive breast cancer following surgery, and its killing potential of monoclonal antibodies is mostly based on antibody‐dependent cytotoxicity mediated by NK cells,^59^ indicating that NK cells play an important part in this immunological process. A study involving 196 patients found that PECS II blockade can enhance the vitality of NK cells after breast cancer surgery and promote smoothly the above immunological process.^55^ PECS II block was found to raise the numbers of CD3+, CD4+, and CD4+/CD8+ T cells in peripheral blood 3 and 24 h after breast cancer surgery in another study,^59^ implying that PECS II blockade can also improve cellular immune activity in patients undergoing surgery.

### 5.2 Study on the therapeutic effect of nerve block on immune diseases

Immunodeficiency has been associated with the emergence of chronic ulcerative colitis.^60^ Zhao and his colleagues presented their findings in 2017: SGB was more successful than sulfasalazine in the treatment of chronic ulcerative colitis, and it had fewer side effects, such as abdominal distension, liver and kidney dysfunction, headache, and vomiting.^61^ Similarly, for the effect of SGB on facial seborrheic dermatitis, Kim reported^62^ firstly a case who had received conventional dermatological treatment for 10 years, unfortunately, the symptoms had not improved obviously. However, after SGB, the condition of the patient was greatly improved.^62^ And Kim speculated that SGB has the advantage to improve blood circulation in the brain to maintain the homeostasis of hormones.^62^ According to one research centering on the treatment of chronic ulcerative colitis, there are two effects of SGB,^63^ one is that it can suppress the chemotaxis of neutrophils, eosinophils, and lymphocytes induced by IL‐8 and relieve inflammatory mediators. Another is that it can improve blood circulation by inhibiting the activity of the sympathetic nervous system, thereby providing patients suffering from chronic ulcerative colitis with immune complexes, which can induce the rapid clearance of inflammatory cytokines and improve the condition.

## 6 THE APPLICATION OF THE NERVE BLOCK TECHNIQUE IN TREATING Posttraumatic STRESS DISORDER

PTSD is a chronic, severe, and debilitating mental illness. Stress disorders and subsequent mental disorders are common among victims. Irritability, difficulty concentrating, and sleep disorders are among the most common symptoms of the former, while anxiety, depression, and loss of functions are of the latter. They have a significant impact on patient's physical and mental health and quality of life, especially in patients suffering from PTSD combined with depression.^64^, ^65^

Lipov et al. presented a case in which SGB was conducted on a patient with PTSD, whose symptoms did not get improvement from medication and psychotherapy but from SGB.^66^ A RCT^67^ showed that the participants receiving SGB had a more significant reduction in the severity of PTSD than those without performed SGB. A large‐sample study involving 166 patients who were active‐duty soldiers but had participated in many wars demonstrated that 70% of patients obtained a 3‐month PTSD relief after a single SGB.^68^ So, SGB may have an excellent effect on treating stress disorders. Furthermore, Lynch revealed his findings after a trial that showed SGB may be administered safely for all kinds of PTSD and lessen PTSD symptoms.^69^ On the influence of SGB, some academics have proposed the following viewpoints. That is, increased sympathetic tone inhibits pineal cell function, followed by secreting low‐level plasma melatonin and causing sleep disturbances, which can, in turn, cause an increase in sympathetic tone.^62^ SGB blocks this sympathetic cycle and restores a normal melatonin rhythm, ultimately helping patients to maintain a normal sympathetic tone and improve corresponding disorder symptoms.^62^, ^70^ The effect of SGB on improving mental disorders after stress disorder is also evident. A patient, with a 2‐year history of suicidal ideation caused by depression, got rid of suicidal ideation after SGB.^71^ The effectiveness of SGB on depression was showcased by regulating the hypothalamus–pituitary–adrenal axis (HPA‐axis) and sympathetic‐adrenal medulla system both in humans and in animal models.^72^, ^73^ Conversely, a conclusion was proposed in Hanling's RCT that SGB had no effect on psychotherapy or pain relief in patients with PTSD and that it could not be applied in clinical practice.^74^ This is contrary to the conclusions of much literature as Table 1. Moreover, two flaws in Hanling's research were directly pointed out. One is that a small sample size of 42 participants and a biased identity stage of participants, with the majority of them transitioning out of military service. Another is that veterans can receive monetary compensation based on the severity of PTSD symptoms when they are discharged from the military, which is the main reason why his study's conclusion differs from other studies.^75^

**Table 1:** Literature on stellate ganglion block for the treatment of posttraumatic stress disorder

Literature	Subject	SGB is effective	Level of SGB	Conclusions
Effect of Stellate Ganglion Block Treatment on Posttraumatic Stress Disorder Symptoms: A Randomized Clinical Trial (2020)	Active‐duty service members	Yes	Right SGB	In this trial of active‐duty service members with PTSD symptoms (at a clinical threshold and subthreshold), two SGB treatments 2 weeks apart were effective in reducing CAPS‐5 total symptom severity scores over 8 weeks
Ultrasound Versus Fluoroscopy for Stellate Ganglion Block: A Cadaveric Study (2021)	Human Cadavers	Maybe	Left and right SGB	While there appeared to be a trend favoring ultrasound guidance, no statistical significance was achieved. This was likely due to this being a limited pilot study. Numerous limitations exist in cadaveric studies, and future investigations should be completed to further study this comparison. That said, the use of the SGB may provide significant relief for patients suffering from PTSD
Utility of Cervical Sympathetic Block in Treating Posttraumatic Stress Disorder in Multiple Cohorts: A Retrospective Analysis (2022)	Military and Civilian	Yes	Right SGB at C6	CSB seems to be an effective treatment for PTSD symptoms irrespective of gender, trauma type, PTSD‐related drug use, suicide attempt, or age
The Use of Stellate Ganglion Block in the Treatment of Panic/Anxiety Symptoms with Combat‐Related Posttraumatic Stress Disorder; Preliminary Results of Long‐Term Follow‐Up: A Case Series (2010)	Active‐duty service soldiers	Yes	Right SGB at C6	Selective blockade of the right stellate ganglion at the C6 level is a safe and minimally invasive procedure that may provide durable relief from PTSD symptoms, allowing the safe discontinuation of psychiatric medications
Stellate Ganglion Block Used to Treat Symptoms Associated with Combat‐Related Posttraumatic Stress Disorder: A Case Series of 166 Patients (2014)	Active‐duty service members	Yes	Right SGB at C6	Selective blockade of the right cervical sympathetic chain at the C6 level is a safe and minimally invasive procedure that may provide durable relief from anxiety symptoms associated with PTSD
The Successful Use of Left‐sided Stellate Ganglion Block in Patients That Fail to Respond to Right‐sided Stellate Ganglion Block for the Treatment of Posttraumatic Stress Disorder Symptoms: A Retrospective Analysis of 205 Patients (2022)	Patients with PTSD	Yes	Left SGB when right SGB was not effective	Based on our sample of 205 patients receiving SGB for PTSD, we concluded that at least 4.4% did not respond to a right‐sided SGB but did have a significant response to a left‐sided SGB
Efficacy of Stellate Ganglion Block in the Treatment of Anxiety Symptoms from Combat‐Related Posttraumatic Stress Disorder: A Case Series (2013)	Patients with PTSD	Yes	Right SGB at C6	Selective blockade of the right stellate ganglion at C6 is a minimally invasive procedure with an excellent safety profile that may provide sustained relief of PTSD symptoms. The procedure may also provide benefit for those who are resistant to psychotropic intervention

## 7 THE APPLICATION OF THE NERVE BLOCK TECHNIQUE IN PROMOTING ENHANCED RECOVERY AFTER SURGERY

The rapid recovery of intestinal function after surgery is one of the goals of ERAS.^76^ Postoperative intestinal exhaust, defecation, and active eating are often used to evaluate postoperative gastrointestinal function recovery. However, PONV, intestinal paralysis, and abdominal distension will delay the recovery of postoperative intestinal function, resulting in a slew of negative consequences, including postoperative pain, lengthened duration of hospital stay, and high diagnostic and treatment costs.^77^

The use of opioids during the perioperative phase is a high‐risk factor for PONV;^78^ therefore, using opioids sparingly can lower the risk of PONV. Several nerve blocks, including the retrolaminar block (RLB),^40^, ^79^ the erector spinae plane block (ESPB),^80^ and the TAPB^81^ have been demonstrated to lower perioperative opioid consumption. As a result, experts recommended using nerve blocks as part of anesthetic management programs to decrease the incidence of PONV, increase patient comfort during medical treatment, and improve the quality of perioperative anesthesia management.^78^ Nerve blocks were also used to treat intestinal paralysis and abdominal distension after surgery. Patients undergoing laparotomies can benefit from a midthoracic epidural block because it allows them to get out of bed earlier for rehabilitation exercises, reducing postoperative intestinal paralysis and abdominal distension.^76^ ESPB was implemented by Gultekin to relieve postoperative pain, extend the time of rescue analgesia, speed up the gastrointestinal function recovery, as well as reduce the times of PONV in patients experiencing percutaneous nephrolithotomy (PCNL).^80^ In addition, TAPB and SGB can promote postoperative intestinal peristalsis, exhaust, defecation, and active eating, minimizing the risk of postoperative intestinal paralysis or infection in patients following surgery.^82^, ^83^ The following are plausible principles of nerve blocks to enhance gastrointestinal function in postoperative patients, according to pertinent studies. First, after blocking sympathetic nerves, the inhibitions of gastrointestinal smooth muscle activity, mucosal secretory function, and vasoconstriction are alleviated, promoting the recovery of gastrointestinal function by enhancing intestinal peristalsis, vasoconstriction, and blood perfusion.^84^ Second, after nerve blocks suppress the sympathetic nerves of the gastrointestinal tract, the excitability of the parasympathetic nerve may reflexively rise to improve gastrointestinal motility, gastric emptying, and other functions.^85^ Third, nerve blocks may attenuate postoperative inflammatory responses and alleviate inflammation‐related gastrointestinal dysfunction.^86^

## 8 CONCLUSIONS AND PROSPECTS

In conclusion, nerve blocks that can help with the treatment or improvement of multi‐system illnesses have been initially used in heart failure, ventricular arrhythmia, PAH, POCD, PTSD, ERAS, and so on. When the medicine's therapeutic effect is not sufficient or drug resistance develops, nerve blocks may be a useful alternative. However, there is currently a paucity of studies on the nonanalgesic effect of nerveblock in different systems, and systematic and targeted research, including large‐sample or multi‐center RCTs, urgently needs to be implemented. Fortunately, ultrasound visualization technology has been created and is widely utilized in clinical and scientific research, allowing for the exact block of tiny nerves to be performed. That is nerve block offers tremendous research potential in the treatment of multi‐system disorders or symptoms.

## AUTHOR CONTRIBUTIONS

Ying Ran collected data, Guang‐Ting Zhang wrote the original draft, Feng‐Lin Wang edited the draft, and De‐Xing Liu supervised the work. All authors have read and agreed to the published version of the manuscript.

## CONFLICT OF INTEREST

The authors declare no conflict of interest.

## TRANSPARENCY STATEMENT

All the authors affirm that this manuscript is an honest, accurate, and transparent account of the study being reported; that no important aspects of the study have been omitted; and that any discrepancies from the study as planned (and, if relevant, registered) have been explained.

### ETHICS STATEMENT

Not Applicable.

## Data Availability

Data sharing is not applicable to this article as no new data were created or analyzed in this study.
